# *Salmonella* infections in Denmark from 2013–2022 with focus on serotype distribution, invasiveness, age, sex, and travel exposition

**DOI:** 10.1007/s10096-024-04808-9

**Published:** 2024-03-21

**Authors:** Nicholas Slinning Aarø, Mia Torpdahl, Torben Rasmussen, Martin Jensen, Hans Linde Nielsen, The Danish Study Group for Enteric Infection has the following members, Ming Chen, Jørgen Engberg, Hanne Marie Holt, Lars Lemming, Lisbeth Lützen, Marc Trunjer Kusk Nielsen, Bente Ruth Scharvik Olesen, Ingrid Maria Cecilia Rubin, Kristian Schønning

**Affiliations:** 1 Department of Clinical Microbiology, Aalborg University Hospital, Aalborg, Denmark; 2 Department for Bacteria, Parasites & Fungi, Statens Serum Institut, Copenhagen, Denmark; 3 Department of Data Integration and Analysis, Statens Serum Institut, Copenhagen, Denmark; 4 Research Data and Biostatistics, Aalborg University Hospital, Aalborg, Denmark; 5 Department of Clinical Medicine, Aalborg University, Aalborg, Denmark

**Keywords:** *Salmonella*, Salmonellosis, Non-typhoid *Salmonella* (NTS), Typhoid *Salmonella*, Invasiveness

## Abstract

**Purpose:**

To analyze the nationwide incidence of *Salmonella* infections in Denmark from 2013 to 2022.

**Methods:**

Confirmed cases of *Salmonella enterica* subsp. *enterica* were examined using the National Register of Enteric Pathogens during 2013–2022. Proportions, incidence rates (IR), relative risk (RR), and 95% confidence intervals (CI) were calculated to assess differences in serotypes, invasiveness, age, sex, and travel exposure.

**Results:**

We identified 9,944 Danish *Salmonella enterica* subsp. *enterica* cases, with an average annual incidence rate of 16.9 per 100,000 inhabitants, declining during the COVID-19 pandemic. Typhoidal cases totaled 206, with an average annual IR of 0.35 per 100,000 inhabitants. Enteric fever patients had a median age of 24 years (IQR:17–36). Leading non-typhoid *Salmonella* (NTS) serotypes were *S.* Enteritidis (26.4%), monophasic *S.* Typhimurium (16.5%), and *S.* Typhimurium (13.5%). Median age for NTS cases was 42 (IQR: 18–62), with even sex distribution, and a third reported travel prior to onset of disease. The overall percentage of invasive NTS (iNTS) infection was 8.1% (CI: 7.6–8.7). Eleven serotypes were associated with higher invasiveness, with *S.* Dublin and *S.* Panama having the highest invasiveness with age and sex-adjusted RR of 7.31 (CI: 6.35–8.43) and 5.42 (CI: 3.42–8.60), respectively, compared to all other NTS serotypes. Increased age was associated with higher RR for iNTS infection.

**Conclusion:**

During the decade, there was a limited number of typhoidal cases. The dominant NTS serotypes were *S.* Enteritidis and monophasic *S.* Typhimurium, whereas *S.* Dublin and *S.* Panama exhibited the highest invasive potential.

## Introduction

*Salmonella*, a genus of Gram-negative bacilli, is a significant global cause of human illness [1]. Its classification relies on antigenic variations in lipopolysaccharides (O-antigens), flagellar proteins (H-antigens), and capsular polysaccharides (Vi-antigens), as outlined in the White-Kauffmann-Le Minor scheme [2]. Among these, *Salmonella enterica* subspecies *enterica* predominantly comprises serotypes responsible for human infections. This subspecies further divides into typhoidal *Salmonella*, which primarily affects humans, and non-typhoidal *Salmonella* (NTS), with zoonotic origins that can be transmitted to humans via animal contact or contaminated food, resulting in Salmonellosis, one of the most common diarrheal diseases worldwide [3].

Typhoidal serotypes, which encompass *S*. Typhi and *S.* Paratyphi (A, B, C), are causing systemic illnesses, commonly referred to as typhoid and paratyphoid fever. These illnesses, collectively known as enteric fever, pose a significant public health challenge, particularly in numerous low- and middle-income countries, especially those with inadequate access to clean water and sanitation systems. However, enteric fever is relatively rare in the EU/EEA and mainly acquired during travel to countries outside the EU/EEA, particularly South Asia, and in 2019 the EU/EEA notification rate was 0.37 cases per 100,000 population years [4].

Salmonellosis is the second most frequently reported foodborne gastrointestinal infection in the EU, surpassed only by Campylobacteriosis, with a notable 87,908 human cases recorded in 2019, corresponding to a notification rate of 19.5 cases per 100,000 population years, whereas the rate subsequently decreased to 13.7 in 2020 and 15.7 in 2021, as reported in The EU One Health 2021 Zoonoses Report [5]. NTS infection often manifest as self-limiting enterocolitis, typically not requiring antibiotic treatment in immunocompetent individuals [3]. However, NTS can breach the intestinal barrier, causing invasive NTS infection (iNTS), which involves the bloodstream and other normally sterile sites [3]. *S.* Enteritidis and *S.* Typhimurium exhibit the highest disease burden, manifesting as both intestinal and extraintestinal infections [3]. In contrast, *S.* Dublin, a bacterium adapted to cattle, predominantly elicits bloodstream infections in humans [6]. Bacteremia rates have been reported to range from 2.7% to 8% [3, 7–12]. Recent studies have also highlighted less common NTS serotypes, such as *S.* Virchow, *S.* Panama, *S.* Poona, *S.* Heidelberg, *S.* Chester, and *S.* Napoli, as potential contributors to iNTS [8–12].

The objectives of this study were to provide insights into the nationwide prevalence of *Salmonella enterica* subspecies *enterica* infections in Denmark from 2013 to 2022, by employing a nationwide population-based cohort design and to further describe the contribution of different serotypes, invasiveness, age and sex distribution, and travel exposure.

## Materials and methods

Our study included 9,944 cases of *Salmonella enterica* subsp. *enterica* (hereafter referred to as *Salmonella*) registered in the National Register of Enteric Pathogens from January 1, 2013, to December 31, 2022 [13]. The register contains information on all confirmed *Salmonella* infections, including data on subspecies and serotypes, as well as details such as the date and type of sampling (stool, urine, blood, etc.), and patient demographics including age, sex, and when reported travel history. In Denmark, Departments of Clinical Microbiology (DCMs) located at 10 major hospitals, perform the primary diagnostics of *Salmonella*. When a DCM identified *Salmonella* to genus level in a clinical specimen (such as stool, urine, or blood culture), the diagnostic result was transmitted to the Danish Microbiology Database, MiBA (https://miba.ssi.dk/). Starting in 2017, all human *Salmonella* data were extracted from MiBa and integrated into the National Register of Enteric Pathogens. Prior to 2017, all cases were reported directly from the DCMs and manually added to the register. Cases are recorded as distinct episodes, meaning that each patient-infectious agent combination is documented only once within any six-month period.

The study population encompassed all Danish inhabitants (numbers were 5,623,501 in 2013, and 5,928,364 in 2022), each possessing a Danish civil registration number.

### Isolation and further characterization of *Salmonella*

Throughout the study period, blood cultures at the DCMs were conducted using automated blood culture systems, namely Bactec™ (BD, Franklin Lakes, NJ, USA) or BacT/Alert (BioMérieux, Marcy l’Etoile, France). The identification of *Salmonella* in clinical samples (including stool, urine, and blood) was typically accomplished through conventional methods, involving the growth of colonies with red pigmentation and, in most cases, a black center (It's worth noting that certain serotypes, such as *S.* Typhi and *S.* Paratyphi A, may not exhibit this black center.). These culture methods employed SSI enteric medium and XLD agar (SSI diagnostica, Hillerød, Denmark), often supplemented by Vitek2 (BioMérieux, Marcy-l'Étoile, France), API 20E (BioMérieux, Marcy-l'Étoile, France), MALDI-TOF (Bruker, Bremen, Germany), and/or agglutination test for *Salmonella* species [14]. Following a *Salmonella* diagnosis at the local DCM, culturable isolates were submitted to Statens Serum Institut for serotyping. Isolates received from 2013 to 2017 were serotyped according to the White-Kauffmann-Le Minor scheme [2] by agglutination with O- and H-antigen specific sera (SSI Diagnostika, Hillerød, Denmark). Serotypes of isolates received from 2018 to 2022 were predicted from whole genome sequencing (WGS). In short, WGS was performed by extracting DNA and preparation using a Promega Wizard genomic DNA purification kit (Promega, Madison, WI) and a Nextera XT v2 DNA library preparation kit (Illumina, San Diego, CA) according to the manufacturers’ protocols. Serotypes were predicted from sequences using an in-house script based on a combination of the Achtman seven gene MLST scheme for *Salmonella* [15] and SeqSero ver. 1.0 [16]. *Salmonella* Typhi and *S.* Paratyphi A, B, and C were classified as typhoidal *Salmonella* cases. However, the d-tartrate fermenting variant of *S.* Paratyphi B, commonly known as *S*. Java, predominantly caused enterocolitis and was therefore categorized as NTS [12].

### Data analysis

Categorical data were presented as absolute numbers and percentages. Contingency tables were generated to display the absolute numbers of different typhoidal and NTS serotypes in relation to age, sex, and source of isolation (invasive vs. non-invasive). Infections caused by *Salmonella* strains obtained from blood, cerebrospinal fluid, peritoneal fluid, pleural fluid, synovial fluid, bone, or other normally sterile sites were categorized as invasive infections. Conversely, *Salmonella* strains isolated from stool, urine, skin, soft tissue abscesses or wounds were designated as non-invasive NTS infections. The term "invasiveness" was employed to quantify the proportion of invasive cases, as defined above, in relation to the total number of cases within a specified group of serotypes. Age was reported as medians with interquartile range [IQR], and further categorized into seven age groups: 0–4, 5–14, 15–24, 25–44, 45–64, 65–84, and ≥ 85 years, with small children aged 0–4 as the reference group. We calculated incidence rates (IR) per 100,000 inhabitants based on the background population and residence in one of the five Danish Administrative Regions (https://www.statistikbanken.dk). For typhoidal *Salmonella* cases, we conducted descriptive analysis, including a Student t-test to compare age distribution among typhoidal strains. For NTS, we additionally estimated the relative risk (RR) using a Poisson model with robust variance estimation by region clusters. We calculated binary RR and 95% confidence intervals (CI) relative to all other NTS serotypes. Travel exposure was simply defined as travel outside of Denmark, as recorded in the surveillance data, regardless of the purpose. Countries were grouped according to the United Nations geoscheme. Stata 18 (College Station, TX: StataCorp LLC) was used for the analysis.

## Results

### Danish *Salmonella* enterica subsp. enterica cases during 2013–2022

There was a total of 9,944 Danish *Salmonella* enterica subsp. enterica cases during 2013–2022, corresponding to a mean annual IR of 16.9 per 100,000 inhabitants. During 2013–2019, the annual IR was unaltered with 18.8 per. 100,000 inhabitants, falling to 10.7 and 12.1 per 100,000 inhabitants during 2020 and 2021, respectively. This decline was followed by an increase in IR to 15.9 per 100,000 inhabitants in 2022. During the entire study period, there was a seasonal peak incidence during in the summer months, whereas the lowest IR was seen during the winter season and spring.

A total of 101 cases were included, with 86 patients having two episodes (36/50 with identical/different serotypes), 10 patients with three episodes (7/3), and 5 patients having four to six episodes (3/2), all occurring more than six-months apart. The entire cohort had a median age of 41 years, (IQR: 18–62 years) and consisted of 4,980 (50%) females and 4,964 (50%) males.

Out of the 9,944 *Salmonella* cases analysed, 616 isolates (6.2%) were excluded because we had no information on serotype. These isolates were obtained from various sources, with 582 from stool, 24 from urine, 7 from blood, and 3 from other sources. The remaining cases, comprising 206 typhoidal *Salmonella* and 9,122 NTS isolates, were included in the study.

### Typhoidal *Salmonella* cases

**Fig. 1:**
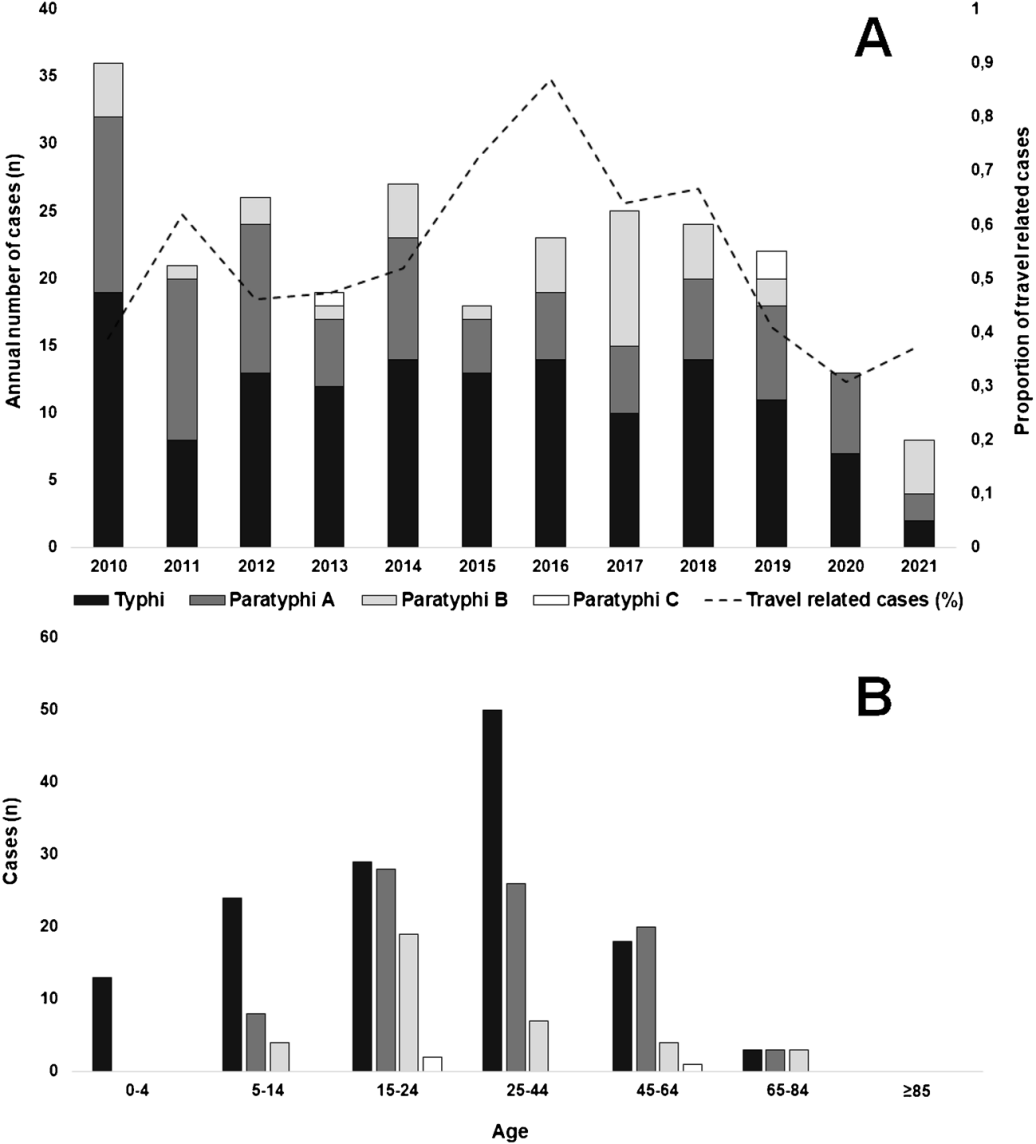
(**A**) Annual number of Danish typhoidal *Salmonella* cases and proportion of travel related cases and (**B**) the distribution per age group of *Salmonella* Typhi and the Paratyphoid serovars in Denmark, 2013–2022

The 206 typhoidal cases were distributed between 116 (56.3%) positive cases of *S.* Typhi, 54 (26.2%) *S.* Paratyphi A, 33 (16.0%) *S.* Paratyphi B, and three (1.5%) *S.* Paratyphi C. Notably, no patient experienced more than one episode of typhoidal infection during the study period. Overall, the average annual IR was 0.35 per 100,000 inhabitants. While the prevalence remained relatively stable until 2020, a noticeable decline occurred in both the number and proportion of travel-related cases during the Covid-19 pandemic, as depicted in Fig. 1A. Two-thirds of all typhoidal cases, were diagnosed in the Capital Region of Denmark, with an average annual IR of 0.72 per 100,000 inhabitants. The remaining one-third of cases exhibited an even geographical distribution across the other four Regions of Denmark, with an average annual IR of 0.16 per 100,000 inhabitants. The sex distribution was approximately equal, comprising 97 (47%) males and 109 (53%) females, and the median age of the affected individuals was 24 years (IQR: 17–36), as presented in Table 1. The highest incidence of most typhoidal cases was observed among young adults. However, *S.* Typhi also accounted for 35 (30%) cases in the age group below 15 years, as depicted in Fig. 1B. The mean age for *S.* Typhi cases was 25.4 years, which was significantly lower than the mean age for all cases of *S.* Paratyphi infection, which was 30.4 years (p-value = 0.04).

**Table 1:** Distribution of typhoidal *Salmonella* cases, Denmark, 2013–2022

Serotype	Sex distribution (Male/Female)	Median age (IQR)	Number of invasive cases (Invasiveness^a^ in %)	Travel related cases^b^
All typhoidal cases (n = 206)	97/109	24 (17–36)	168 (81.6%)	114 (55.3%)
*S.* Typhi (n = 116)	53/63	23 (11–35)	99 (85.3%)	61 (52.6%)
*S.* Paratyphi A (n = 54)	24/30	21 (19.5–29)	46 (85.2%)	30 (55.6%)
*S.* Paratyphi B (n = 33)	19/14	26.5 (20–46)	23 (69.7%)	20 (60.6%)
*S.* Paratyphi C (n = 3)	1/2	-	0	3 (100%)

^a^Absolute number and proportion of invasive cases to the total number of cases

^c^Absolute number and proportion of cases with international travel history prior to infection

Regarding the source of specimens, 168 (81.6%) cases were characterized as invasive primarily originating from blood, with *S.* Typhi and *S.* Paratyphi A both displaying the highest invasiveness rates at 85.3% (CI: 78.9%-91.8%) and 85.2% (CI: 75.7%-94.7%), respectively, as shown in Table 1. Almost all isolates from non-invasive typhoidal cases were derived from stool samples, with only one isolate originating from urine.

Throughout the study period, 114 (55.3%) cases were linked to known travel exposure in the registry, all of which had a history of travel outside Europe. In contrast, travel exposure information was unavailable for 82 (39.8%) cases, and ten (4.9%) cases had no documented travel history before their infection. Among the cases with travel-related exposure, Southern Asia (n = 59), particularly Pakistan (n = 32) and India (n = 20), emerged as the most frequent travel destinations. This was followed by Southeastern Asia (n = 22), Latin America (n = 17), Africa (n = 8), and Western Asia (n = 8).

### Non-typhoid *Salmonella* Cases

**Fig. 2:**
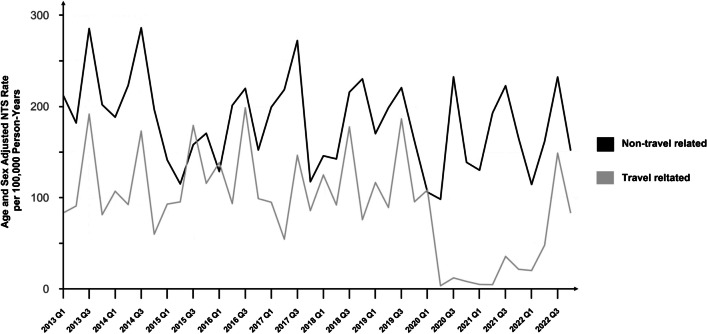
Age and sex adjusted Non-Typhoidal Salmonellosis (NTS) cases (n = 9,122) with or without travel exposure in Denmark, presented by quarters, 2013–2022

The prevalence of NTS cases remained relatively stable until the onset of the Covid-19 pandemic in Denmark in early spring 2020, after which a notable decrease was observed. Specifically, there were only 570 and 625 reported NTS cases during 2020 and 2021, respectively, as illustrated in Fig. 2. A distinct seasonal pattern was also observed in NTS cases, characterized by a peak incidence during the third quarter, with the highest number of cases recorded in August. Conversely, there was a notable decrease in NTS cases during December while, January exhibited the highest number of cases during the winter and spring season. In contrast to typhoidal cases, there was a more equally distributed mean incidence rate of NTS cases across the five Danish regions, lowest for the Central Denmark Region (12.8 per. 100,000 inhabitants), and highest for the Capital Region and the North Denmark Region (both, 16.3 per. 100,000 inhabitants). The overall median age for NTS cases was 42 (IQR: 18–62). The sex distribution was even with 4,570 (50%) males and 4,552 (50%) females of all the cases.

The specimen sources were distributed as follows: Among the non-invasive NTS cases, there were a total of 8,382 cases, with 8,099 originating from stool samples, 240 from urine samples, 11 from swabs (with no information regarding the anatomical site), and 32 from other non-invasive sources. In contrast, there were 740 cases of invasive NTS infections (see below), including 706 cases detected through blood culture, 19 through cerebrospinal fluid analysis, and 15 through other means such as bone biopsy, synovial fluid, peritoneal fluid, and pleural fluid.

Two-thousand four-hundred and four (26.4%) of all NTS cases were caused by *S.* Enteritidis, followed by 1,508 (16.5%) cases of the monophasic variant of *S.* Typhimurium (O:4,5,12; H:i:- and O:4,12; H:i:-), and 1,230 (13.5%) cases of *S.* Typhimurium. Other serotypes, with more than 100 total cases during the study period are shown in Table 2.

**Table 2:** This table includes the prominent non-typhoidal *Salmonella* (NTS) serotypes, each with over one hundred reported total cases in Denmark from 2013 to 2022, alongside those exhibiting heightened invasiveness levels. Counts, sex distribution, age, and binary relative risks (RR) are shown

Serotype	Total number of cases	Sex distribution Male/female	Median age (IQR)	Number of iNTS cases (Invasiveness)^a^	Unadjusted RR (95% CI)	Adjusted RR^b^ (95% CI)
All NTS	9,122	4,570/4,552	42 (18–62)	740^c^ (8.1%)	-	
Enteritidis	2,404	1,261/1,143	37 (13–59)	169 (7.0%)	0.83 (0.73–0.96)	0.92 (0.81–1.04)
monophasic *S.* Typhimurium^d^	1,508	797/711	46 (17–65)	55 (3.6%)	0.41 (0.29–0.57)	0.38 (0.28–0.53)
Typhimurium	1,230	593/637	40 (15–61)	74 (6.0%)	0.71 (0.58–0.86)	0.74 (0.60–0.90)
Newport	250	120/130	53 (30–66)	12 (4.8%)	0.54 (0.36–0.81)	0.47 (0.32–0.69)
Stanley	234	120/114	30 (13–52)	12 (5.1%)	0.63 (0.31–1.25)	0.78 (0.38–1.58)
Dublin	233	129/104	69 (60–77)	166 (71.2%)	10.99 (9.77–12.37)	7.31 (6.35–8.43)
Infantis	201	103/98	41 (22–60)	6 (3.0%)	0.36 (0.23–0.57)	0.36 (0.23–0.58)
Java^e^	151	78/73	29 (20–53)	16 (10.6%)	1.30 (0.94–1.80)	1.58 (1.13–2.20)
Agona	133	60/73	29 (18–55)	3 (2.3%)	0.28 (0.07–1.08)	0.32 (0.09–1.18)
Virchow	132	66/66	33 (20–53)	16 (12.1%)	1.52 (0.86–2.68)	1.80 (1.08–3.01)
Derby	110	49/61	63 (42–75)	3 (2.7%)	0.33 (0.10–1.12)	0.25 (0.07–0.83)
Kentucky	109	50/59	46 (24–59)	1 (0.9%)	0.11 (0.01–1.06)	0.11 (0.01–1.03)
Braenderup	106	46/60	48 (21–70)	5 (4.7%)	0.59 (0.28–1.24)	0.53 (0.27–1.02)
Oranienburg	103	41/62	48 (27–64)	9 (8.7%)	1.08 (0.57–2.07)	1.03 (0.59–1.81)
O:4,5,12; H:b:-^f^	82	39/43	28 (18–55)	12 (14.6%)	1.83 (1.26–2.66)	2.21 (1.48–3.30)
Chester	77	38/39	39 (19–60)	14 (18.2%)	2.25 (2.04–2.49)	2.35 (2.10–2.63)
Napoli	56	29/27	34 (18–58)	12 (21.4%)	2.47 (1.41–4.32)	2.72 (1.46–5.07)
Javiana	47	27/20	37 (13–55)	11 (23.4%)	2.90 (2.60–3.23)	3.21 (2.44–4.22)
Heidelberg	36	13/23	25 (15–62)	7 (19.4%)	2.46 (1.26–4.81)	2.92 (1.36–6.28)
Panama	32	14/18	42 (14–60)	12 (37.5%)	4.80 (3.20–7.22)	5.42 (3.42–8.60)
Muenster	28	16/12	30 (15–50)	5 (17.9%)	2.20 (1.04–4.62)	2.75 (1.32–5.72)
Schwarzengrund	26	14/12	33 (19–52)	5 (19.2%)	2.37 (1.46–3.84)	2.91 (1.73–4.89)
Other	1,862	883/979	44 (20–63)	120 (6.4%)	0.76 (0.59–0.97)	0.73 (0.59–0.92)

^a^Proportion of invasive NTS cases to the total number of NTS cases

^b^Adjusted for age and sex

^c^Blood culture = 706, Cerebrospinal fluid = 19, Other (bone biopsy, synovial fluid, peritoneal fluid, pleural fluid) = 15

^d^Including O:4,5,12; H:i:- and O:4,12; H:i:-

^e^In this report the d-tartrate fermenting variant *S.* enterica subspecies enterica serovar Paratyphi B is referred to as *S.* Java

^f^O:4,5,12; H:b:- is a monophasic variant *S.* Java

### Invasive NTS cases

The highest prevalence of iNTS infection was seen in 2013 (n = 96) and lowest in 2020 (n = 52). Overall, the mean invasiveness for NTS was 8.1% (CI: 7.6%-8.7%), see Table 2. Among the iNTS serotypes, the most prevalent were *S.* Enteritidis (n = 169; 22.8%), *S.* Dublin (n = 166; 22.4%), *S.* Typhimurium (n = 74; 10.0%), and monophasic *S.* Typhimurium (n = 55; 7.4%). Apart from *S.* Dublin, which exhibited a consistent incidence throughout the entire study period, all the aforementioned serotypes displayed diminished incidences in 2020 and 2021.

When assessing invasiveness among all serotypes in relation to their total cases, we found that *S.* Dublin, *S.* Panama, *S*. Javiana, *S*. Heidelberg, *S*. Schwarzengrund, *S.* Muenster, *S.* Napoli, *S*. Chester, the monophasic variant of *S*. Paratyphi B or *S*. Paratyphi B var. (O:4,5,12; H:b:-), *S.* Virchow, and *S.* Java exhibited significantly higher adjusted RR for invasiveness compared to all other NTS serotypes combined. *Salmonella* Dublin showed the highest risk of iNTS infection with adjusted RR of 7.31 (CI: 6.35–8.43) when compared to all other NTS. In contrast, *S*. Derby, *S*. Infantis, monophasic *S*. Typhimurium, *S.* Newport, and *S.* Typhimurim had low adjusted RR for iNTS infection, see Table 2.

Overall, the male sex was associated with higher RR for iNTS infection by 1.29 (CI: 1.12–1.48). Additionally, the RR of iNTS infection showed an upward trend with increasing age, as outlined in Table 3. Specifically, the RR of iNTS was 2.24 (1.84–2.72) for individuals aged 45–64 years, 4.86 (4.15–5.69) among those aged 65–84 years, and 6.47 (4.70–8.89) for those aged 85 years and above, compared to small children (reference group). Furthermore, *S.* Dublin cases had a noticeable higher median age of 69 years (IQR: 60–77 years) compared to all other NTS cases, as mentioned above.

**Table 3:** Number of non-typhoidal *Salmonella* (NTS), sex distribution, invasive NTS (iNTS) cases, and travel related cases for non-iNTS and iNTS, as well as relative risk (RR) for iNTS, for each defined age group, Denmark, 2013–2022

Age group	Total NTS cases	Sex distribution Male/female	iNTS cases (%^a^)	Male/Female iNTS cases (%)	Travel Related non-iNTS (%)^b^	Travel related iNTS (%)^c^	RR
All	9,122	4,570/4,552	740 (8.1%)	417 (56.4%) / 323 (43.6%)	3,105 (37%)	145 (19.6%)	-
0–4	1,090	559/531	37 (3.4%)	23 (62.2%) / 14 (37.8%)	339 (32.2%)	13 (35.1%)	1.00
5–14	893	502/391	28 (3.1%)	16 (57.1%) / 12 (42.9%)	397 (46.0%)	10 (31.3%)	0.93 (0.64–1.34)
15–24	1,149	548/601	58 (5.0%)	30 (51.7%) / 28 (48.3%)	518 (47.5%)	29 (50.9%)	1.47 (1.00–2.16)
25–44	1,600	821/779	89 (5.6%)	46 (51.7%) / 43.3 (48%)	590 (39.0%)	34 (38.2%)	1.61 (1.34–1.93)
45–64	2,385	1,187/1,198	181 (7.6%)	103 (56.9%) / 78 (43.1%)	882 (40.0%)	38 (21.2%)	2.24 (1.84–2.72)
65–84	1,812	878/934	304 (16.8%)	175 (57.6%) / 129 (42.4%)	376 (24.9%)	21 (6.9%)	4.86 (4.15–5.69)
≥ 85	193	78/115	43 (22.3%)	24 (55.8%) / 19 (44.2%)	3 (2.0%)	0	6.47 (4.70–8.89)

^a^Proportion of iNTS cases to the remaining number of NTS cases for the defined age group

^b^Number and proportion of non-iNTS cases for the defined age group with recent international travel history

^c^Number and proportion of iNTS cases for the defined age group with recent international travel history

### NTS and travel exposure

Among all NTS cases, travel exposure was reported in 3,250 (35.6%) instances, while 3,505 (38.4%) cases were of domestic origin with no travel exposure, and finally travel information was missing in 2,367 (26.0%) of the cases. Notably, there was a higher RR of iNTS infection for individuals with missing travel data or of domestic origin compared to cases with documented travel exposure (RR: 2.27, CI: 1.90–2.71).

Regarding NTS serotypes linked with invasive infections, a notably high proportion of travel-related cases was evident in *S.* Kentucky, *S.* Stanley, *S.* Java and its monophasic variant (O:4,5,12; H:b:-), *S.* Enteritidis, *S.* Virchow, *S.* Panama, *S.* Muenster, and *S.* Heidelberg. In these serotypes, at least half of the cases were associated with travel exposure. Conversely, the lowest proportions of travel-related cases were identified in *S.* Dublin, *S.* Napoli, and *S.* Derby, as outlined in Table 4.

**Table 4:** Number of travel related cases and associated travel destination for the non-typhoidal *Salmonella* (NTS) serotypes, each with over one hundred reported total cases in Denmark from 2013 to 2022, alongside those exhibiting heightened invasiveness levels

Serotype	Travel related cases (%)^a^	Domestic origin (%)	Unknown (%)^b^
All NTS	3,250 (35.6%)	3,505 (38.4%)	2,367 (26.0%)
Enteritidis	1,365 (56.8%)	539 (22.4%)	500 (20.8%)
Monophasic *S.* Typhimurium^c^	254 (16.8%)	870 (57.7%)	384 (25.5%)
Typhimurium	256 (20.8%)	669 (54.4%)	305 (24.8%)
Newport	84 (33.6%)	100 (40.0%)	66 (26.4%)
Stanley	143 (61.1%)	41 (17.5%)	50 (21.4%)
Dublin	6 (2.6%)	80 (34.3%)	147 (63.1%)
Infantis	52 (25.9%)	83 (41.3%)	66 (32.8%)
Java^d^	89 (58.9%)	33 (21.9%)	29 (19.2%)
Agona	49 (36.8%)	58 (43.6%)	26 (19.6%)
Virchow	73 (55.3%)	21(15.9%)	38 (28.8%)
Derby	8 (7.3%)	66 (60.0%)	36 (32.7%)
Kentucky	73 (67.0%)	13 (11.9%)	23 (21.1%)
Braenderup	37 (34.9%)	40 (37.7%)	29 (27.4%)
Oranienburg	22 (21.4%)	55 (53.4%)	26 (25.2%)
O:4,5,12; H:b:-^e^	41 (50.0%)	19 (23.2%)	22 (26.8%)
Chester	32 (41.6%)	25 (32.5%)	20 (25.9%)
Napoli	4 (7.1%)	31 (55.4%)	21 (37.5%)
Javiana	19 (40.4%)	8 (17.0%)	20 (42.6%)
Heidelberg	19 (52.8%)	8 (22.2%)	9 (25%)
Panama	17 (53.1%)	7 (21.9%)	8 (25.0%)
Muenster	15 (53.6%)	6 (21.4%)	7 (25.0%)
Schwarzengrund	12 (46.2%)	5 (19.2%)	9 (34.6%)
Other	595 (32.0%)	734 (39.4%)	533 (28.6%)

^a^Number and proportion of cases with international travel history prior to infection

^b^Number and proportion of cases where travel history was missing

^c^Including O:4,5,12; H:i:- and O:4,12; H:i:-

^d^In this report the d-tartrate fermenting variant *S.* enterica subspecies enterica serovar Paratyphi B is referred to as *S.* Java

^e^O:4,5,12; H:b:- is a monophasic variant *S.* Java

Noteworthy travel destinations by regions and sub-regions included Southeastern Asia (n = 882; Thailand = 508, Indonesia = 172, Vietnam = 89, Philippines = 44, Other = 69), Europe (n = 794; Spain = 197, Greece = 126, Italy = 59, Other = 412), Western Asia (n = 681; Turkey = 593, Other = 88), Africa (n = 574; Egypt = 210, Other = 364), Southern Asia (n = 164; India = 68, Sri Lanka = 40, Pakistan = 31, Other = 25), and Latin America and the Caribbean (n = 114). During 2020 and 2021, the annual number of travel-related cases dropped to 111 (19.7%) and 57 (9.1%), respectively.

## Discussion

In this comprehensive analysis spanning from 2013 to 2022, we identified nearly ten thousand cases of *Salmonella enterica* subsp. enterica in Denmark. Before the onset of the COVID-19 pandemic, the incidence remained stable. However, during the pandemic, we observed a decline in cases. By 2022, the incidence had nearly returned to pre-pandemic levels. Typhoidal cases were limited in number, with the highest incidence recorded in the Capital Region and a predilection among children and young adults. The most prevalent NTS serotypes were *S.* Enteritidis, monophasic *S.* Typhimurium, and *S*. Typhimurium. Patients with NTS infections had a median age in their early forties, with an even distribution of sex. Moreover, we identified a substantial percentage (8.1%) of iNTS infections among all NTS cases. Eleven serotypes exhibited a higher adjusted RR of invasive infection compared to other NTS serotypes, with *S.* Dublin and *S.* Panama displaying the highest invasiveness. Risk factors associated with iNTS infection included increased age and male sex. Lastly, it's worth noting that approximately one-third of all NTS cases reported a history of travel exposure.

Turning our attention to typhoidal strains, we observed an overall invasiveness rate of more than eighty percent consistent with their well-documented ability to cause systemic illness. In Denmark, the annual number of typhoidal cases, primarily associated with travel to endemic regions, has remained relatively stable since 1995, with only a few domestically acquired cases reported [17]. The Danish incidence rate of typhoidal fever, at 0.35 per 100,000 inhabitants, closely mirrors the incidence rate in the EU [4]. Up until 2020, the prevalence of typhoidal cases displayed a consistent pattern. Nonetheless, a noticeable reduction in prevalence and the relative proportion of travel-related cases was observed, likely influenced by the impacts of the Covid-19 pandemic and the subsequent implementation of travel restrictions.

In our cohort, four out of five cases presented with bacteremia, with the highest rates observed in *S.* Typhi and *S.* Paratyphi A infections. In contrast, data from the Canadian National Enteric Surveillance Program (NESP) covering the years 2006 to 2019 reported a bacteremia rate of 35.2% for typhoidal strains, significantly lower compared to the findings in the Danish dataset [10].

While transmission typically occurs through fecal–oral routes, most cases in developed countries are acquired abroad by travelers returning from typhoid-endemic regions. Our registry data revealed that 55.3% of typhoidal cases were linked to travel outside Europe, with travelers returning from Pakistan, India, Southeastern Asia, Latin America, and, to a lesser extent, Africa, and Western Asia. The data of travel exposure in the registry was lower than expected, as travel information was unavailable for more than a third of cases. Nevertheless, we also identified ten typhoid cases that were domestically acquired. This may include transmission within a household or infection of healthcare professionals who handle typhoid fever patients or their samples.

As for the rest of the EU, there was also a significant decrease in NTS cases in Denmark during the COVID-19 pandemic, and infections with NTS also displayed a distinct seasonal pattern, with the highest incidence during the third quarter, particularly in August, whereas January also saw a surge, likely due to increased travel during the holiday season. A seasonal peak in August has been observed for decades in Denmark, but a Danish population-based study indicated that the influence of seasonal variation diminishes with increased severity of NTS infection, including NTS bacteremia [18].

Historically, *S.* Enteritidis and *S.* Typhimurium have consistently accounted for two-thirds of all Danish zoonotic *Salmonella* infections during the 1980s, 1990s, and 2000s [19]. However, the annual number of cases has steadily declined since the mid-1990s, primarily due to the successful eradication of *S.* Enteritidis in domestic poultry [20]. In the period from 2013 to 2022, *S.* Enteritidis represented more than one-fourth of all cases, and when combined with *S.* Typhimurium and its monophasic variant, these three serotypes collectively constituted 56.4% of all Danish NTS cases. This closely mirrors the trends observed in Europe, where these three serotypes account for approximately two-thirds of all reported cases [5]. These findings underscore the consistency of NTS serotype prevalence between Denmark and the broader European context, emphasizing the relevance of regional data in the larger epidemiological landscape.

In contrast to typhoidal cases, NTS infections were more evenly distributed across Danish regions. The Central Denmark Region recorded the lowest incidence, while the Capital Region and the North Denmark Region reported the highest rates. These regional disparities may, in part, arise from various factors, such as differences in diagnostic methodologies, travel patterns, and consequent exposure to NTS. Additionally, variations in the rural versus urban composition between regions may also have played a role in the observed differences.

The median age for individuals affected by NTS was 42 years, and there was an even distribution of cases between males and females. Our analysis identified increased age as a significant risk factor for iNTS infections, in line with findings from prior studies that older adults and males are more likely to develop iNTS infection [8, 9]. The reasons behind this gender difference warrant further investigation, but it may be associated with different behaviors related to food consumption and hygiene practices. Due to the absence of clinical data or other registry information, including International Classification of Diseases, 10th revision (ICD-10) diagnoses, we were unable to draw definitive conclusions regarding the attribution of age-related comorbidities. Katiyo et al. demonstrated a close association between NTS bacteremia and hospitalization with specific comorbidities, including cardiovascular, pulmonary, and genitourinary conditions, as well as medical conditions such as diabetes, hypertension, and acute kidney failure [8].

We identified a high number of invasive NTS infections compared to recent data from England, Holland, Italy, Canada, and Australia [8–12]. This remained true even when considering only patients with bacteremia and including *Salmonella* cases identified only to the genus level (7.3%, CI: 6.8–7.9) in the denominator. Eleven serotypes exhibited elevated adjusted RR for iNTS infection rates: *S.* Dublin, *S.* Panama, *S.* Javiana, *S.* Heidelberg,* S*. Schwarzengrund, *S.* Muenster, *S.* Napoli,, *S.* Chester, *S.* Java and its monophasic variant, and *S.* Virchow. Notably, *S.* Dublin demonstrated the highest invasiveness compared to all other NTS serotypes.

*S.* Dublin is a host-restricted bacterium primarily found in cattle, with transmission to humans occurring primarily through the consumption of unpasteurized dairy products and beef [21]. Ongoing efforts have aimed at eradicating *S.* Dublin from Danish cattle farms since 2002, leading to a reduction in the number of Danish milk-producing farms testing positive for *S.* Dublin [22]. However, while the bacterium persists in the cattle population, Danish cattle will remain a known reservoir for *S.* Dublin, facilitating transmission to humans through food products. *S.* Dublin accounted for 2.6% of all NTS cases and 22% of all iNTS infections in Denmark. Notably, the incidence of *S.* Dublin remained stable throughout the study period, unaffected by the pandemic.

The reported invasiveness of *S.* Panama is also documented in other developed countries [8–10]. In contrast, the invasiveness of *S.* Javiana in Denmark was not reported in the Canadian dataset [10], where the case count (n = 1,384) significantly exceeded ours (n = 47). Due to our smaller sample size, there is a possibility that our study may have led to a potential overestimation of the invasiveness of *S.* Javiana.

Human cases with *S.* Napoli were previously uncommon in Europe, however, during the last twenty years the prevalence of cases has been increasing, particularly in central Europe [23]. There is no known zoonotic reservoir of *S.* Napoli, and the source of human infection is often unknown [23, 24]. Still, outbreaks have been associated with various food products like imported chocolate in England and ham in Italy [25, 26]. An analysis made in 2018 by Sabbatucci et al. also suggests that contaminated surface water and ready-to-eat vegetables irrigated with contaminated surface water are notable sources of foodborne *S.* Napoli cases [24].

We also identified *S.* Virchow as having higher RR for invasive infection, but only in the adjusted analysis. A recent study from Queensland, Australia, reported the notification rate from 2007 through 2016, and found that *S.* Virchow accounted for 25% of all iNTS isolates, while *S.* Choleraesuis, *S.* Dublin, and *S.* Panama having the highest invasiveness index [12]. Despite this, *S.* Choleraesuis was remarkably rare in absolute numbers, with only six cases (four iNTS and two non-iNTS), equating to 0.2 per thousand cases in total. This aligns with our data, where we identified only two isolates during our 10-year study period.

Overall, approximately one third of all NTS cases in our study were related to travel, similar to findings in other Nordic countries [27]. Most serotypes in our report, associated with iNTS infection were associated with travel. However, *S.* Typhimurium and its monophasic variants, as well as *S.* Napoli and *S.* Dublin were not frequently related to travel. Although *S.* Typhimurium and its monophasic variants were among the most prevalent serotypes in our study, travel exposure was only found in approximately one fifth of the cases, indicating that most of the cases are of domestic origin, in line with previously reported findings from Denmark [20]. Transmission of *S.* Typhimurium to humans typically occurs through consumption of contaminated pork meat or pork meat produce. Nonetheless, in 2020 Statens Serum Institut reported an outbreak of *S.* Typhimurium in Denmark that originated from Psyllium-seeds, showing that the source attribution for *S.* Typhimurium is diverse [28].

This study has inherent limitations due to its reliance on passive surveillance data from Denmark's healthcare system. Notably, milder cases of *Salmonella* infections, which often resolve without medical intervention, may not be diagnosed and thus go unaccounted for in our analysis. Consequently, our findings are based on the subset of more severe *Salmonella* cases. Additionally, the lack of comprehensive travel exposure data, particularly for typhoidal *Salmonella* cases, poses a significant limitation. Moreover, 6.2% of *Salmonella* cases were excluded due to missing serotype, but it could be plausible that these cases were evenly distributed among the different serotypes, potentially having minimal impact on the estimates. A major strength of the study is the comprehensive and complete data for age, sex, and specimen type, which was 100% complete.

In conclusion, our 10-year analysis of *Salmonella* infections in Denmark identified nearly 10,000 cases. Incidence remained stable before the COVID-19 pandemic, experienced a decline during the pandemic, and nearly recovered by 2022. Typhoidal cases were limited, primarily affecting the Capital Region, and particularly children and young adults. We identified a substantial number (8.1%) of iNTS infections among all NTS cases, with *S*. Dublin and *S*. Panama exhibiting the highest invasiveness. Risk factors for iNTS infection included increased age and male sex. Approximately one-third of NTS cases reported a history of travel exposure. These findings provide critical insights into the epidemiology of *Salmonella* in Denmark, including incidence trends, prevalent serotypes, and key risk factors associated with invasive illness.

## Data Availability

Data can be shared if deemed relevant by contacting the Corresponding Author directly.
